# Tamoxifen reduces fat mass by boosting reactive oxygen species

**DOI:** 10.1038/cddis.2014.553

**Published:** 2015-01-08

**Authors:** L Liu, P Zou, L Zheng, L E Linarelli, S Amarell, A Passaro, D Liu, Z Cheng

**Affiliations:** 1 Department of Human Nutrition, Foods and Exercise, Fralin Life Science Institute, College of Agriculture and Life Science, Virginia Tech, Blacksburg, VA, USA

## Abstract

As the pandemic of obesity is growing, a variety of animal models have been generated to study the mechanisms underlying the increased adiposity and development of metabolic disorders. Tamoxifen (Tam) is widely used to activate Cre recombinase that spatiotemporally controls target gene expression and regulates adiposity in laboratory animals. However, a critical question remains as to whether Tam itself affects adiposity and possibly confounds the functional study of target genes in adipose tissue. Here we administered Tam to Cre-absent forkhead box O1 (FoxO1) floxed mice (f-FoxO1) and insulin receptor substrate Irs1/Irs2 double floxed mice (df-Irs) and found that Tam induced approximately 30% reduction (*P*<0.05) in fat mass with insignificant change in body weight. Mechanistically, Tam promoted reactive oxygen species (ROS) production, apoptosis and autophagy, which was associated with downregulation of adipogenic regulator peroxisome proliferator-activated receptor gamma and dedifferentiation of mature adipocytes. However, normalization of ROS potently suppressed Tam-induced apoptosis, autophagy and adipocyte dedifferentiation, suggesting that ROS may account, at least in part, for the changes. Importantly, Tam-induced ROS production and fat mass reduction lasted for 4–5 weeks in the f-FoxO1 and df-Irs mice. Our data suggest that Tam reduces fat mass via boosting ROS, thus making a recovery period crucial for posttreatment study.

Excess fat mass or adiposity is the hallmark of obesity, the rapidly growing epidemic.^1, 2^ In the United States, over two-thirds of adults are overweight or obese according to the statistics of years 2011–2012.^3^ For children, the overweight or obese population accounts for about 25% in the 2–5-year olds and 33% in school students (including adolescents).^3^ It is estimated that obesity care accounts for 21% of national healthcare expenditures, that is, 190.2 billion US dollars per year, in the United States.^4^ Because adipose tissue is an important endocrine organ, which secretes adipokines or cytokines that regulate inflammatory responses and metabolic homeostasis, aberrant adiposity dysregulates adipokine levels and leads to a variety of metabolic disorders and complications, such as diabetes and cardiovascular diseases.^5^ As such, the healthcare burden that obesity imposes on the society is far greater.

To understand the molecular mechanism of obesity development, various rodent models have been generated to study the gain or loss of functions of different genes.^6, 7^ To this end, the Cre/lox site-specific recombination system has been versatile to generate conditional mouse mutants, controlling gene expression and activity in target tissues.^8, 9^ In particular, Tamoxifen (Tam) is used to activate Cre recombinases spatiotemporally *in vivo* through intraperitoneal (I.P.) or subcutaneous administration.^10, 11, 12^ Injection of Tam at a dose of 1–8 mg/kg body weight for 5 consecutive days deletes target genes, thus establishing a versatile system to study functional genes in obesity.^8, 9, 10, 11, 12^

The use of Tam in clinical treatments has led to the argument about its potential effect on body fat or weight gain in human patients.^13, 14^ It raises the question as to whether and how Tam influences adipocytes and fat mass in the experimental animal models after administration. Exclusion of direct regulation of adiposity by Tam as a confounding factor in animal models is critical to better understand target genes in adipogenesis and metabolic homeostasis. In the present work, we present the evidence that 5-day administration of Tam significantly reduces mouse fat mass, which persists till weeks 4–5 after the treatment. At the cellular level, Tam promotes the production of reactive oxygen species (ROS), which is accompanied with enhanced apoptosis, autophagy and adipocyte dedifferentiation. However, treatment of adipocytes with antioxidant *N*-acetyl cysteine (NAC) dramatically counteracted Tam-induced ROS and suppressed apoptotic and autophagic markers, concomitant with reversal of adipocyte dedifferentiation. *In vivo*, fat mass was restored upon the normalization of ROS, which is associated with suppressed adipocyte dedifferentiation and downregulated apoptotic and autophagic markers. Our data reveal a ROS-mediated mechanism by which Tam induces fat mass reduction. As it may confound the posttreatment study, deliberate determination of the recovery period after Tam administration is essential to understand the functions of target genes using Tam-induced knockout mice.

## Results

### Tam induced fat mass reduction in mice

**Figure 1:**
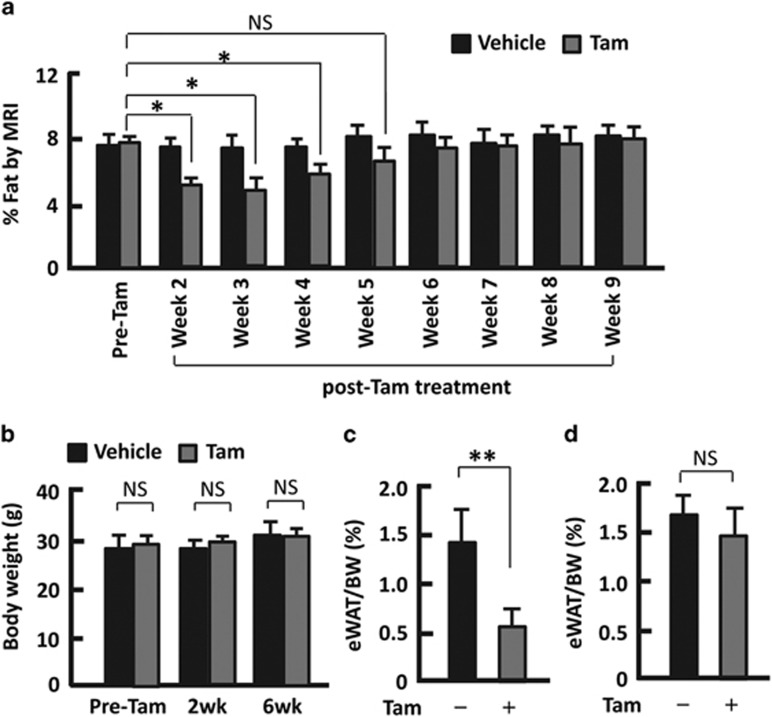
Tam reduced fat mass in f-FoxO1 mice. (**a**) The kinectics of fat mass regulation after 5-day administration of Tam. (**b**) Measurement of body weight before Tam treatment (pre-Tam), 2 weeks (2wk) and 6 weeks (6wk) after Tam injection. (**c**) The weight of epididymal adipose tissue (eWAT) at week 2 in mice treated with Tam. (**d**) The weight of eWAT at week 6 in mice treated with Tam. *n*=4–6; *P<0.05; **P<0.01; NS, not significant

To test the effect of Tam on fat mass, we conducted a 5-day I.P. administration of Tam (1 mg/20 g body weight) on forkhead box O1 (FoxO1) floxed mice bearing no Cre recombinase (f-FoxO1),^15, 16, 17^ by following a standard protocol established previously.^12^ Two weeks after Tam administration, the body fat was reduced by 34% (*P*<0.05) in f-FoxO1 mice (Figure 1a). To validate the findings, we treated insulin receptor substrate 1 (Irs1) and Irs2 double floxed mice without Cre recombinase (df-Irs) in a similar way,^15, 16^ and found that fat mass was also significantly reduced (26%, *P*<0.05; Supplementary Figure S1A). However, the changes in body weight were insignificant between vehicle (sunflower oil) control and the treatment groups (Figure 1b; Supplementary Figure S1B). Monitoring of the kinetics of fat mass change suggested that the reduction was persistent till week 5 (week 4 in df-Irs mice), after which the fat percentage was comparable to the pretreatments (Figure 1a; Supplementary Figure S1A). In line with this finding, the weight of epididymal white adipose tissue (eWAT) was significantly reduced in Tam-treated f-FoxO1 mice at week 2, while there was no significant difference at week 6 (Figures 1c and d). By contrast, injection of the vehicle caused indiscernible change in the body fat mass (Figure 1a; Supplementary Figure S1A). Therefore, the reduction of fat mass in mice arose primarily from Tam treatment. Given that both mouse models shared this phenotype, we used f-FoxO1 mice for the following mechanistic study.

### Tam promoted apoptosis and autophagy in adipose tissue

**Figure 2:**
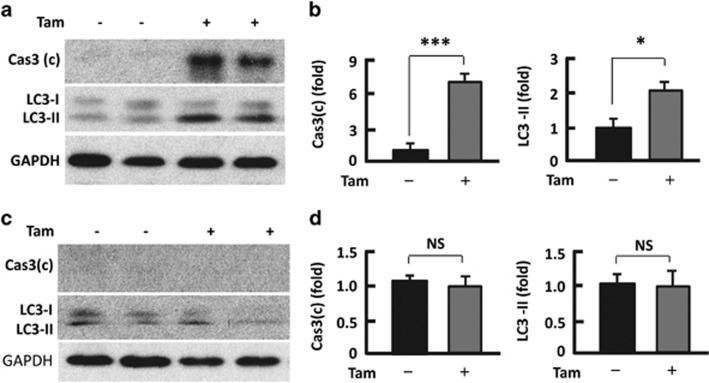
Tam increased apoptotic and autophagic regulators in adipose tissue. (**a** and **b**) At week 2 after Tam administration, western blotting (**a**) was performed to analyze Cas3(c) and LC3 with densitometric analysis (**b**) of western blotting images using the NIH ImageJ software; *n*=5–7. (**c** and **d**) At week 6 after Tam administration, western blotting (**c**) was performed to analyze Cas3 (c) and LC3, and densitometric analysis (**d**) of western blotting images with the NIH ImageJ software; *n*=5–7. GAPDH (glyceraldehyde 3-phosphate dehydrogenase) was probed as a loading control. *P<0.05; ***P<0.0001; NS, not significant

The regulators of apoptosis and autophagy were implicated in the regulation of fat mass.^18, 19, 20, 21, 22, 23^ To examine whether Tam had effects on apoptosis and autophagy, we used eWAT at weeks 2 and 6 after Tam administration and analyzed the mediators of apoptosis and autophagy—the activated or cleaved caspase 3 (Cas3(c)) and microtubule-associated protein 1A/1B-light chain 3-phosphatidylethanolamine conjugate (LC3), respectively.^24, 25^ As shown in Figures 2a and b, Tam treatment increased the level of Cas3(c) by 6.8-fold (*P*<0.0001) and the autophagosomal marker LC3-II by 1.9-fold (*P*<0.05) at week 2. However, these changes were largely reversed at week 6 and showed no statistical significance (Figures 2c and d).

### Tam promoted the production of ROS

**Figure 3:**
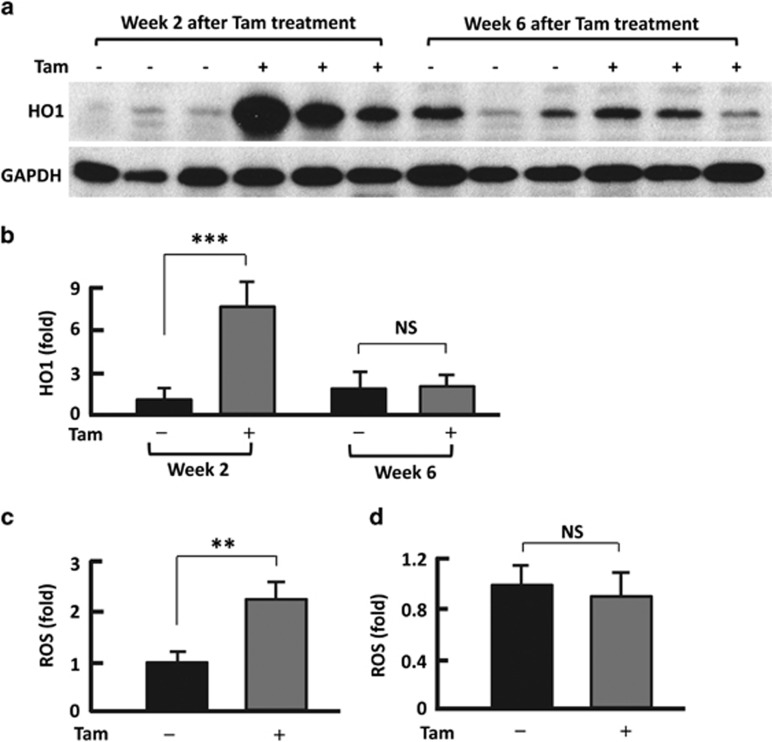
Tam promoted ROS and oxidative stress. (**a**) Western blotting analysis of HO1 in mouse adipose tissues at weeks 2 and 6, respectively, after Tam administration, with GAPDH (glyceraldehyde 3-phosphate dehydrogenase) probed as a loading control. (**b**) Densitometric analysis of western blotting images using the NIH ImageJ software; *n*=6–8. (**c**) Measurement of ROS in adipose tissue at week 2 after Tam administration (*n*=3–4). (**d**) Measurement of ROS in adipose tissue at week 6 after Tam administration (*n*=3–4). **P<0.01; ***P<0.0001; NS, not significant

ROS and the resultant oxidative stress have an important role in apoptosis and autophagy.^26, 27, 28^ To examine whether ROS and oxidative stress was involved in Tam-induced effects, we analyzed heme oxygenase 1 (HO1), the sensitive indicator of cellular oxidative stress.^29^ Tam treatment upregulated HO1 protein levels in adipose tissue by over 7-fold (*P*<0.0001) in mice at week 2; however, at week 6, the HO1 abundance in the treated mice was comparable to that in untreated mice, showing no statistical significance (Figures 3a and b). Interestingly, the HO1 levels in untreated mice were higher at week 6 than at week 2, supporting the notion that HO1 expression increases with age.^30, 31^ Measurement of ROS levels in adipose tissue indicated a 2.3-fold elevation in Tam-treated mice than in vehicle-treated mice at week 2 (Figure 3c), but they became statistically insignificant at week 6 (Figure 3d). Of note, the upregulation and downregulation of ROS and HO1 levels seems to coincide well with the changes in Cas3(c), LC3-II and fat mass (Figures 1, 2, 3).

### Antioxidant abolished Tam-induced ROS, apoptosis and autophagy

**Figure 4:**
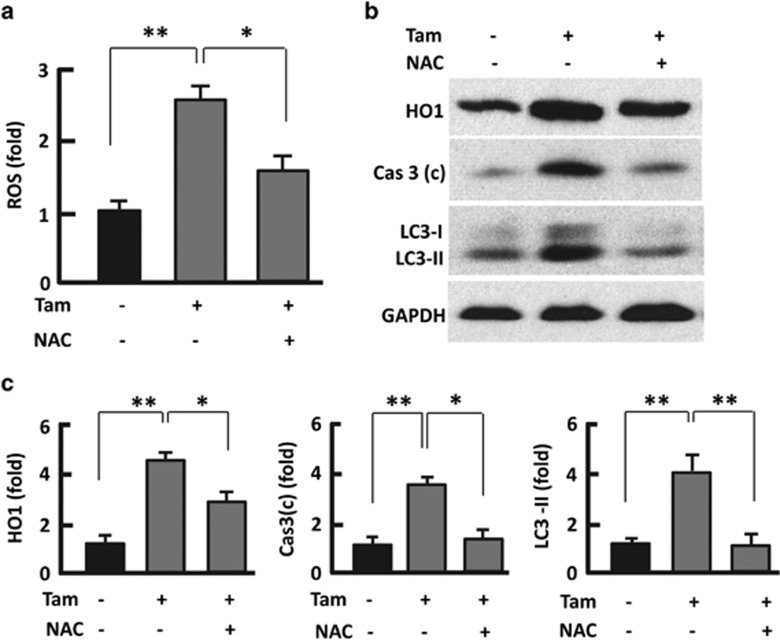
Antioxidant NAC attenuated ROS level and reversed Tam effects. (**a**) Measurement of ROS in 3T3L1 adipocytes. (**b** and **c**) Western blotting analysis (**b**) of HO, Cas3 (c) and LC3 in 3T3L1 adipocytes after 48-h treatment with Tam (128 *μ*M) or Tam (128 *μ*M) plus NAC (1 mM), with densitometric analysis (**c**) of western blotting images using the NIH ImageJ software; GAPDH (glyceraldehyde 3-phosphate dehydrogenase) was probed as a loading control. *n*=3–5; *P<0.05; **P<0.01

To map the interaction between ROS and other Tam-induced cellular events, we treated 3T3L1 adipocytes with Tam combined with a potent ROS-scavenger NAC.^32, 33^ As observed in adipose tissue, Tam induced significant upregulation of HO1 in 3T3L1 adipocytes, and the effect was dose dependent in the tested range of 0–128 *μ*M (Supplementary Figure S2). Tam also promoted ROS production by 2.5-fold (*P*<0.01) in 3T3L1 adipocytes (Figure 4a) and significantly upregulated the apoptosis regulators Cas3(c) and autophagosomal marker LC3-II (Figures 4b and c). However, inclusion of NAC in the treatments suppressed Tan-induced elevation of ROS and HO1 and normalized the protein levels of Cas3(c) and LC3-II (Figures 4a–c).

### Antioxidant reversed Tam-induced reduction in cell density and adipocyte population

**Figure 5:**
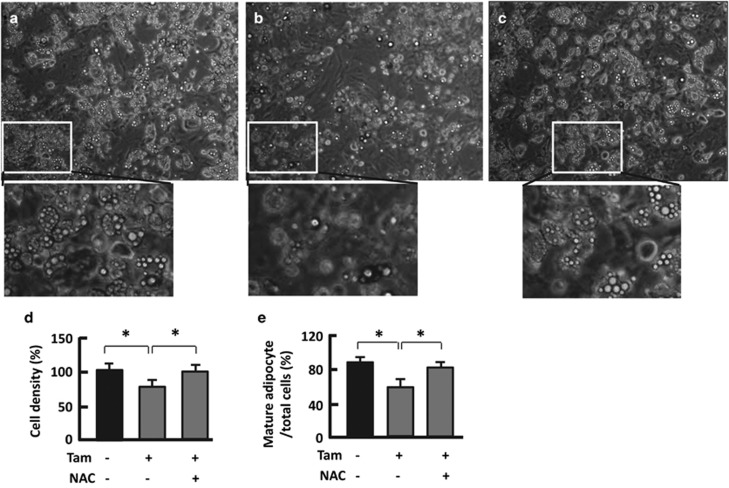
Antioxidant NAC mitigates Tam effect on cell density and mature adipocyte population. (**a**–**c**) Microscopy imaging of 3T3L1 adipocytes treated with vehicle (**a**), 128 *μ*M Tam (**b**) and Tam (128 *μ*M) plus NAC (1 mM) (**c**). The microscope was set at × 100. (**d** and **e**) Measurement of cell density and population of mature adipocytes using the NIH ImageJ software; *n*=6–8. *P<0.05

Because alteration in adipocyte number affects adiposity,^2^ we asked whether Tam treatment influenced cell density. Compared with the vehicle-treated adipocytes, Tam-treated cells showed a significant decrease in cell density (24%, *P*<0.05), consistent with the upregulation of apoptotic marker (Figures 5a, b and d, Figures 4b and c). Interestingly, the population of lipid-droplet-containing cells (i.e., mature adipocytes) also declined (36%, *P*<0.05), implying a process of ‘dedifferentiation' might be induced by Tam (Figures 5a, b and e).^34, 35, 36^ Regardless, addition of the ROS-scavenger NAC largely restored the cell density and population of mature adipocytes (Figures 5c and e).

### Antioxidant reversed Tam-induced downregulation of peroxisome proliferator-activated receptor gamma (PPARγ)

**Figure 6:**
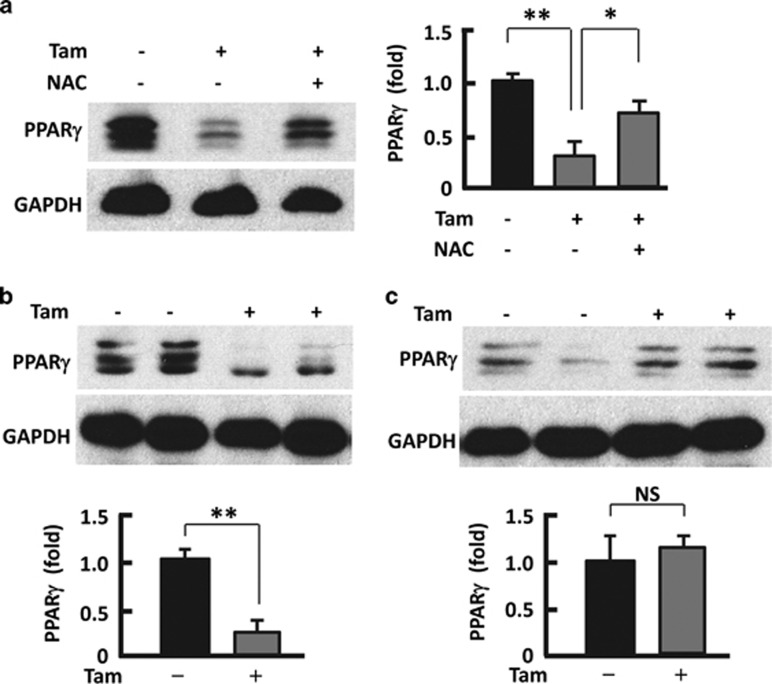
Counteracting or normalizing ROS reduced Tam effect on PPAR*γ*. (**a**) Western blotting analysis (left panel) of PPAR*γ* in 3T3L1 adipocytes after 48-h treatment with Tam (128 *μ*M) or Tam (128 *μ*M) plus NAC (1 mM), with densitometric analysis (right panel) of western blotting images using the NIH ImageJ software; *n*=3–5. (**b**) At week 2 after Tam administration, western blotting (upper panel) was performed to analyze PPAR*γ*, with densitometric analysis (lower panel) of western blotting images using the NIH ImageJ software; *n*=3–5. (**c**) At week 6 after Tam administration, western blotting (upper panel) was performed to analyze PPAR*γ*, with densitometric analysis (lower panel) of western blotting images using the NIH ImageJ software; *n*=3–5. GAPDH (glyceraldehyde 3-phosphate dehydrogenase) was probed as a loading control. *P<0.05; **P<0.01

PPAR*γ* is a key regulator of adipogenesis (*de novo* generation of mature adipocytes) and adipocyte dedifferentiation.^34, 35, 37^ The observation of reduced population of mature adipocytes after Tam treatment prompted us to analyze the effect of Tam on PPARγ. As shown in Figure 6a, PPAR*γ* protein levels were reduced by 74% (*P*<0.01) in Tam-treated adipocytes. However, co-treatment of the adipocytes with Tam and NAC significantly restored PPAR*γ* level. These data suggest that Tam may regulate adipogenesis or population of mature adipocytes through ROS-mediated downregulation of PPAR*γ*. Consistent with this hypothesis, we found that the PPAR*γ* levels were dramatically decreased (69%, *P*<0.01) in the adipose tissues of Tam-treated mice at week 2 (Figure 6b), which is accompanied by elevation of ROS and HO1 (Figures 3a–c). At week 6 when ROS and HO1 levels returned to normal (Figures 3a, b and d); however, the abundance of PPAR*γ* also returned to the values comparable to those in the control mice (Figure 6c). In addition, the reversal of ROS overproduction and PPAR*γ* suppression was accompanied by normalization of fat mass at week 6 (Figures 1, 3 and 6 Supplementary Figure S1).

## Discussion

Tam has been widely used to activate inducible Cre recombinase and knockout target genes in mechanistic studies of adipose development and metabolic homeostasis.^8, 11, 12, 38, 39, 40, 41^ However, the effect of Tam administration on adipocytes and adipose tissue has not been investigated to the best of our knowledge. In this study, we chose to use f-FoxO1 bearing no Cre recombinase for Tam treatment, aiming to rule out the effect of Cre activation (or gene deletion) on fat mass. We found that a 5-day administration of Tam led to a significant reduction of fat mass in mice, which lasted for 4–5 weeks after the last injection. The findings were validated in Cre-absent df-Irs mice. The Tam-induced fat mass reduction could confound the effects of gene knockout, making it critical to allow for 6 weeks as a recovery period before further study is conducted. Note that the recovery period may vary with different animal models, thus warranting a deliberate determination for a specific laboratory model to establish a reliable experimental system.

The mechanism by which Tam reduces fat mass includes several cellular events. Tam treatment increased apoptosis and autophagy, the processes that reduce adipocyte number and have been implicated in adipose regulation.^2, 18, 19, 20, 21, 22, 23^ Indeed, the cell density and population of mature adipocytes decreased after Tam treatment. Tam also promoted adipocyte dedifferentiation and ROS production, whereas normalization of ROS level markedly mitigated Tam-induced adipocyte dedifferentiation, apoptosis and autophagy, concomitant with restoration of mature adipocyte population and fat mass. Together, our data strongly suggest that the short-term (5-day) treatment with Tam reduces fat mass via boosting ROS production.

Tam was shown to induce ROS and oxidative stress in breast cancer cells, hepatoblastoma cells, retinal cells and platelets through activation of NAD(P)H oxidase, the enzyme that also promotes ROS production in macrophages.^32, 42, 43, 44, 45, 46^ The ROS-boosting effect of Tam was extended and further validated by our study in adipocytes and adipose tissues. Importantly, we found that ROS elevation resulted in PPAR*γ* downregulation and adipocyte dedifferentiation, which support the notion that mature adipocytes undergo dedifferentiation under stress conditions.^34, 35^ It was shown that proinflammatory adipocytokines (e.g., TNF*α*) could promote adipocyte dedifferentiation through downregulation of PPARγ.^34, 35^ Given that ROS elevation or oxidative stress increases TNF*α* production,^47^ Tam may promote adipocyte dedifferentiation by activating a ROS–TNF*α*–PPAR*γ* axis. To this end, macrophage infiltration in adipose tissue might have a role, because these phagocytes were shown to instigate ROS and TNF*α* production and also respond sensitively to ROS- and TNF*α*-mediated signaling cascades.^46, 48^

The effect of Tam on fat mass in humans, for example, breast cancer patients, remains inconclusive. Although Tam was reported to increase fat mass through its anti-estrogenic effect,^13^ recent studies loosened the conclusion by showing that Tam has no effect on the fat mass in breast cancer patients.^14^ It should be noted that the Tam dosage and treatment duration for mice in this study significantly differs from that for the long-term Tam treatment of breast cancer patients. To activate Cre recombinase to knock out target genes, animal models are typically treated for 5 consecutive days (administration of 1–8 mg/20 g body weight or 50–400 mg/kg body weight, once a day).^8, 9, 10, 11, 12^ However, Tam therapy for breast cancer patients in the United States generally lasts 5 years, with a dose of 20 mg (either one 20 mg tablet or two 10 mg tablets) taken by month once a day.^49, 50, 51^ Assuming that the body weight of breast cancer patients ranges from 50 kg to 80 kg, the average daily use of Tam would be 0.25–0.4 mg/kg, a dosage being 0.06–0.8% of that used in animal models. Owing to different dosage and treatment duration, the effect of Tam on mouse fat mass observed in this study might not be phenocopied in breast cancer patients with Tam therapy.

## Materials and Methods

### Materials

Dulbecco's modified Eagle's (DMEM) medium was from Corning Inc. (Manassas, VA, USA). Fetal bovine serum (FBS) was from GeneMate (Kaysville, UT, USA). Dexamethasone, 3-isobutyl-1-methylxanthine (IBMX), rosiglitazone and Tam were purchased from Cayman chemical (Ann Arbor, MI, USA). Penicillin/streptomycin (P/S) was from GE Healthcare Life Sciences HyClone Laboratories (Logan, UT, USA). Insulin and NAC were from Sigma-Aldrich (St. Louis, MO, USA). Phosphate-buffered saline (PBS) was from Caisson Laboratories, Inc. (North Logan, UT, USA).

### Mice

The FoxO1 floxed mice (f-FoxO1) and Irs1/Irs2 double floxed mice (df-Irs) were bred and housed as previously described.^15, 16, 52^ Briefly, the mice were housed in plastic cages on a 12-h light–dark photocycle, with free access to water and regular chow diet. Before Tam treatment experiments, male mice (14–16-week old) were weighed, and fat mass was measured with a Bruker Minispec LF90 NMR Analyzer (Bruker Optics, Billerica, MA, USA). Then the mice were transferred to a biosafety level 2 (BSL2) room and administered with Tam (1 mg/20 g body weight) or the vehicle (sunflower oil) by I.P. injection (once a day for 5 consecutive days). After Tam administration, the cages were changed every 2 days until week 2, when the mice were transferred into BSL1 room, and the measurement of body fat mass was resumed. Depending on the experimental design, the mice were weighed and killed to harvest tissue for snap freezing in liquid nitrogen, at week 2 or week 6 after Tam treatment. All the procedures followed the NIH guideline and were approved by the Virginia Tech Institutional Animal Care and Use Committee.

### Cell culture and treatment

3T3L1 preadipocytes (ATCC CL-173, Manassas, VA, USA) were cultured in basal media (i.e., DMEM media supplemented with 10% FBS, 100 units/ml penicillin and 100 *μ*g/ml streptomycin (1 × P/S)), at 37 °C in a humidified atmosphere of 5% CO_2_. The media were replaced every 2 days. Differentiation of 3T3L1 cells was induced as described previously with minor modifications.^53^ Briefly, 3T3L1 cells were grown to confluence (day 0) and maintained in fresh basal media (BMI) for 2 days (days 1–2). At the end of day 2, BMI medium was changed to differentiation medium I (DMI): DMEM supplemented with 10% FBS, P/S (1 × ), IBMX (0.5 mM), dexamethasone (1 *μ*M), insulin (1 *μ*g/ml), and rosiglitazone (2 *μ*M). At the end of day 4, DMI medium was changed to differentiation medium II (DMII): DMEM supplemented with 10% FBS, P/S (1 × ), and insulin (1 *μ*g/ml). At the end of day 6, DMII medium was changed to basal media (BMII), and the cells were maintained in BMII (replaced with fresh basal medium every 2 days) until fully differentiated (day 12). As a control, preadipocytes were maintained in BMI till day 12 and supplied with fresh medium every other day. Upon full differentiation, 3T3L1 adipocytes were treated with Tam for 48 h at the concentrations of 0, 8, 16, 32, 64 and 128 *μ*M and the vehicle 0.1% sunflower as a treatment control.^12^ When applicable, NAC was added at a concentration of 1 mM with Tam for a 48-h treatment to study the role of ROS.^32^ Images of the cells were captured on day 12 with a Nikon ECLIPSE TS100 microscope (Melville, NY, USA), and the cell counting and population analysis was conducted with the NIH ImageJ software (Bethesda, MD, USA).

### ROS measurement

ROS in adipocytes and adipose tissue was measured as previously described,^54, 55^ with a cell-permeable dye 5,6-carboxy-2′,7′-dichlorofluorescein diacetate (Carboxy-DCFDA, Molecular Probes, Grand Island, NY, USA). Snap-frozen adipose tissues were weighed and transferred into buffered medium (5 mmol/l HEPES in PBS) for quick thawing to improve the probe diffusion. After rapid thawing, the medium was discarded. Samples were exposed to 8 *μ*M Carboxy-DCFDA in fresh medium and were incubated at 37 °C for 45 min under agitation. Medium was then removed, and samples were further incubated in a lysis buffer (0.1% SDS, Tris-HCl, pH 7.4) for 15 min at 4 °C. After homogenization, samples were centrifuged at 16 000 × *g* for 20 min at 4 °C. Supernatants were collected and subjected to fluorescence analysis at 530 nm under excitation at 485 nm using a Synergy H4 Hybrid Multi-Mode Microplate Reader (BioTek Instruments, Winooski, VT, USA).

To measure ROS in 3T3L1 adipocytes, 1–5 × 10^6^ cells were harvested with typsin and washed three times with cold PBS, followed by incubation with 8 *μ*M Carboxy-DCFDA in fresh medium (5 mmol/l HEPES in PBS) and were incubated at 37 °C for 45 min under agitation. Medium was then removed, and samples were further incubated in PLC lysis buffer:^15, 52^ (30 mM Hepes, pH 7.5, 150 mM NaCl, 10% glycerol, 1% Triton X-100, 1.5 mM MgCl_2_, 1 mM EGTA, 10 mM NaPPi, 100 mM NaF, 1 mM Na_3_VO_4_) supplemented with protease inhibitor cocktail (Roche, Branchburg, NJ, USA) and 1 mM PMSF for 15 min at 4 °C. After homogenization, samples were centrifuged at 16 000 × *g* for 20 min at 4 °C. Supernatants were collected and subjected to fluorescence analysis at 530 nm under excitation at 485 nm, and the total protein was determined with DC protein assay (Bio-Rad, Hercules, CA, USA) on a Synergy H4 Hybrid Multi-Mode Microplate Reader (BioTek Instruments, Inc.). The ROS levels were normalized to the total protein for each cell dish.

### Western blotting

To prepare tissue lysates, snap-frozen adipose tissues were weighed and homogenized with a Bullet Blender (Next Advance, Averill Park, NY, USA) in PLC lysis buffer supplemented with protease inhibitor cocktail (Roche), 1 mM PMSF, 10 *μ*M TSA (Trichostatin A, Selleckchem, Houston, TX, USA) and 5 mM Nicotinamide (Alfa Aesar, Ward Hill, MA, USA).^15, 52^ For cell lysates, the 3T3L1 adipocytes were washed with ice-cold PBS and homogenized with a Bullet Blender. Total protein concentrations of the lysates were determined using the DC protein assay (Bio-Rad). Western blotting and image analysis were conducted as described previously.^15^ Antibody catalog numbers and vendors are as follows: cleaved caspase-3 Rabbit mAb (9664) and LC3B antibody (no. 2775) from Cell Signaling Technology (Beverly, MA, USA); PPAR-gamma antibody (MA5-14889) and GAPDH antibody (MA5-15738) from Pierce (Rockford, IL, USA) or Thermo Fisher Scientific (Waltham, MA, USA); and HO1 antibody (3391-100) from Biovision (Milpitas, CA, USA).

### Statistical analyses

All results are expressed as means±S.D. and are analyzed by analysis of variance to determine *P* values; *P*<0.05 was considered statistically significant.

## Abbreviations

Carboxy-DCFDA: 5,6-carboxy-2′,7′-dichlorofluorescein diacetate
Cas3(c): cleaved caspase 3
df-Irs: Irs1/Irs2 double floxed mice
eWAT: epididymal white adipose tissue
FoxO1: forkhead box O1
f-FoxO1: FoxO1 floxed mice
HO1: heme oxygenase 1
Irs: insulin receptor substrate
I.P.: intra-peritoneal
LC3: microtubule-associated protein 1A/1B-light chain 3-phosphatidylethanolamine conjugate
NAC: *N*-acetyl cysteine
PPAR*γ*: peroxisome proliferator-activated receptor gamma
ROS: reactive oxygen species
Tam: Tamoxifen
