# Supplementary figures and images for: Tamoxifen reduces fat mass by boosting reactive oxygen species
Source: Cell Death Dis. 2015 Jan 8;6(1):e1586–. doi: 10.1038/cddis.2014.553 (PMC4669751; doi:10.1038/cddis.2014.553)

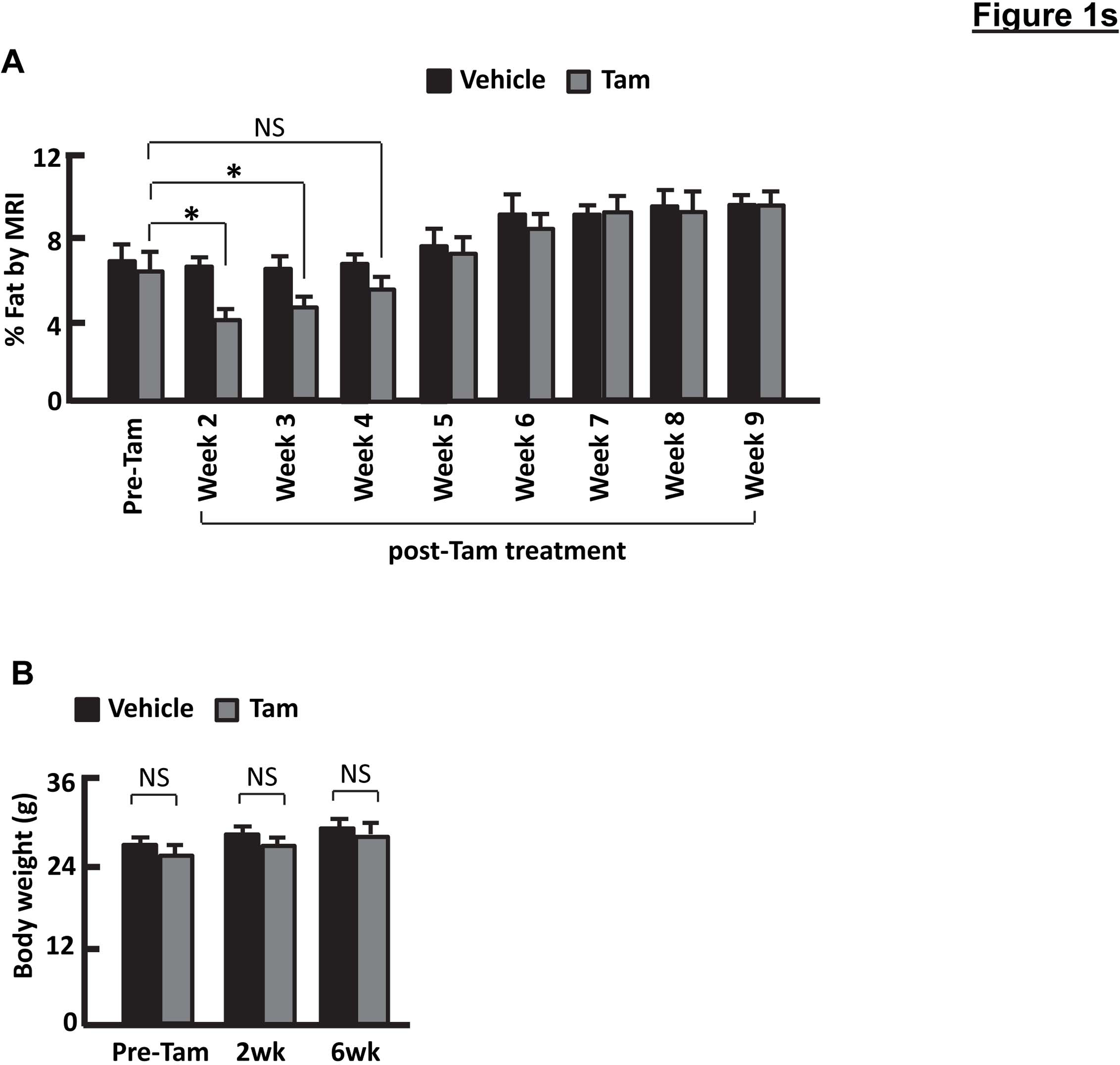

Supplement: Supplementary Figure S1 [file cddis2014553x1.tif]

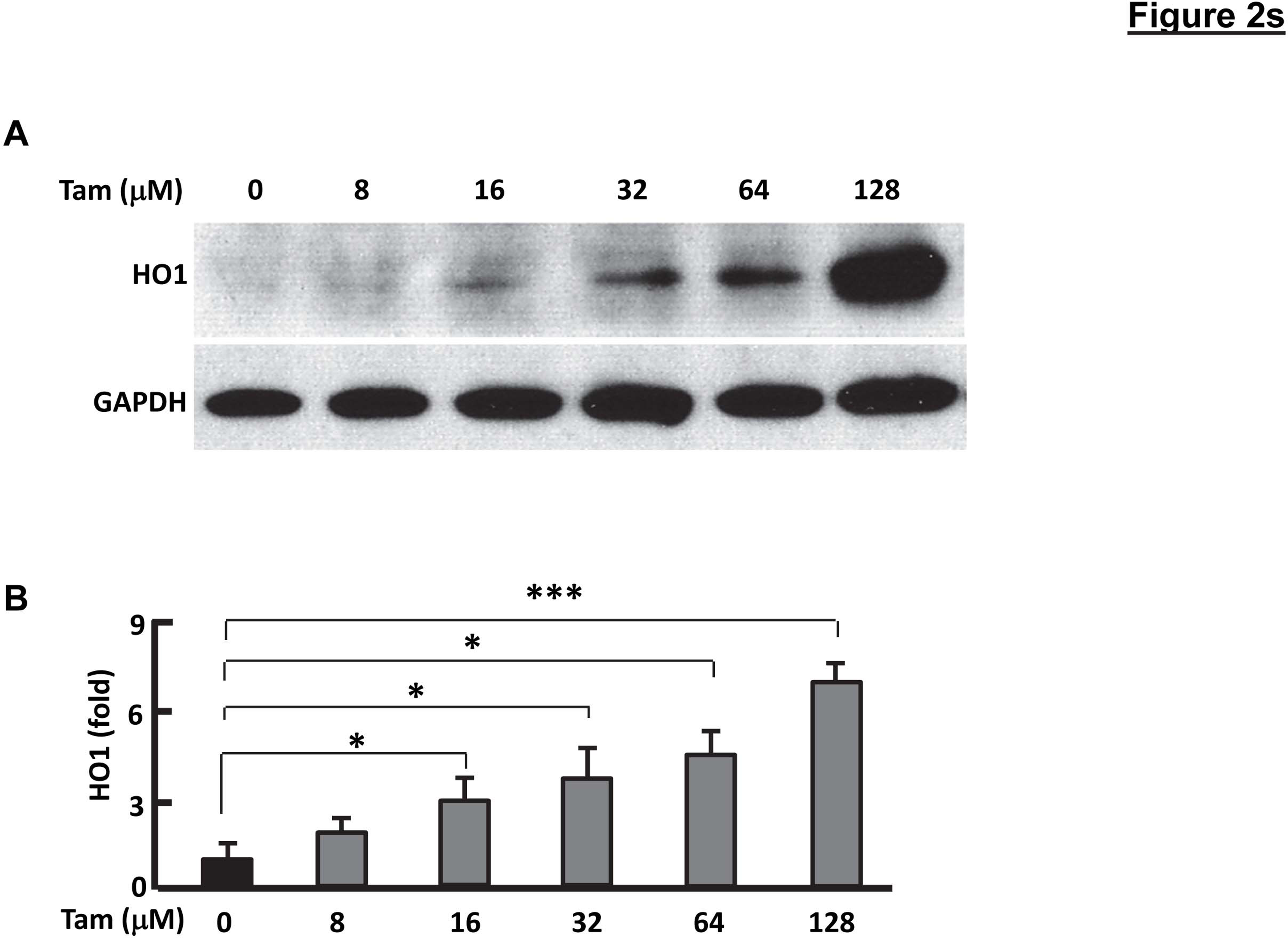

Supplement: Supplementary Figure S2 [file cddis2014553x2.tif]
