# Supplementary material for: Tamoxifen reduces fat mass by boosting reactive oxygen species
Source: Cell Death Dis. 2015 Jan 8;6(1):e1586–. doi: 10.1038/cddis.2014.553 (PMC4669751; doi:10.1038/cddis.2014.553)
Supplement: Supplementary Figure Legends [file cddis2014553x3.doc]

**Figure 1s.** Tam reduced fat mass in df-Irs mice. (A) The kinetics of fat mass regulation after 5-day administration of Tam. (B) Measurement of body weight before Tam treatment (pre-Tam), 2 weeks (2 wk) and 6 weeks (6 wk) after Tam injection. *, p<0.05; NS, not significant.

**Figure 2s.** Tam induced oxidative stress in 3T3L1 adipocytes. Cells were lysed after 48-hour treatment with Tam at various concentrations, and the cell lysates underwent western blot (panel A) for HO1 measurement, with densitometric analysis (panel B) of western blot images using NIH ImageJ software; n=3-5. GAPDH was probed as a loading control. *, p<0.05; ***, p<0.0001.
